# Adapting Evidence-Based Falls Prevention Programs for Remote Delivery — Implementation Insights through the RE-AIM Evaluation Framework to Promote Health Equity

**DOI:** 10.1007/s11121-023-01519-z

**Published:** 2023-04-10

**Authors:** Marlana J. Kohn, Kelly A. Chadwick, Lesley E. Steinman

**Affiliations:** Health Promotion Research Center, University of Washington, Seattle, USA

**Keywords:** Evidence-based programs, Evaluation, RE-AIM, Adaptation

## Abstract

COVID-19 disproportionally impacted the health and well-being of older adults—many of whom live with chronic conditions—due to their higher risk of dying and being hospitalized. It also created several secondary pandemics, including increased falls risk, sedentary behavior, social isolation, and physical inactivity due to limitations in mobility from lock-down policies. With falls as the leading cause of preventable death and hospitalizations, it became vital for in-person evidence-based falls prevention programs (EBFPPs) to pivot to remote delivery. In Spring 2020, many EBFPP administrators began re-designing programs for remote delivery to accommodate physical distancing guidelines necessitated by the pandemic. Transition to remote delivery was essential for older adults and persons with disabilities to access EBFPPs for staying healthy, falls and injury free, out of hospitals, and also keeping them socially engaged. We collaborated with the Administration on Community Living (ACL), the National Council on Aging (NCOA), and the National Falls Prevention Resource Center (NFPRC), for an in-depth implementation evaluation of remotely delivered EBFPPs. We examined the process of adapting and implementing four EBFPPs for remote delivery, best practices for implementing the programs remotely within the RE-AIM evaluation framework. This enhances NFPRC’s ongoing work supporting dissemination, implementation, and sustainability of EBFPPs. We purposively sampled organizations for maximum variation in organization and provider type, geographic location, and reach of underserved older populations (Black, Indigenous, or other People of Color (BIPOC), rural, disabilities). This qualitative evaluation includes provider-level data from semi-structured interviews (*N* = 22) with program administrators, staff, and leaders. The interview guide included what, why, and how adaptations were made to EBFPP interventions and implementation strategies using Wiltsey-Stirman (2019) adaptations framework (FRAME), reach, and implementation outcomes (acceptability, feasibility, fidelity, and costs; Proctor et al., 2011), focusing on equity to learn for whom these programs were working and opportunities to address inequities. Findings demonstrate remote EBFPPs made planned and fidelity-consistent adaptations to remote delivery in partnership with researchers and community organizations, focusing on participant safety both in program content and delivery. Supports using and accessing technology were needed for delivery sites and leaders to facilitate engagement, and improved over time. While remote EBFPP delivery has increased access to EBFPPs for some populations from the perspective of program administrator, leaders, and staff (e.g., caregivers, rural-dwellers, persons with physical disabilities), the digital divide remains a barrier in access to and comfort using technology. Remote-delivered EBFPPs were acceptable and feasible to delivery organizations and leaders, were able to be delivered with fidelity using adaptations from program developers, but were more resource intensive and costly to implement compared to in-person. This work has important implications beyond the pandemic. Remote delivery has expanded access to groups traditionally underserved by in-person programming, particularly disability communities. This work will help answer important questions about reach, accessibility, feasibility, and cost of program delivery for older adults and people with disabilities at risk for falls, those living with chronic conditions, and communities most vulnerable to disparities in access to health care, health promotion programming, and health outcomes. It will also provide critical information to funders about elements required to adapt EBFPPs proven effective in in-person settings for remote delivery with fidelity to achieve comparable outcomes.

COVID-19 disproportionally impacted the health and well-being of older adults—many of whom live with chronic conditions—due to their higher risk of dying and being hospitalized. It also created several secondary pandemics, including increased falls risk, sedentary behavior, social isolation, and physical inactivity due to limitations in mobility from lock-down policies (Hoffman et al., 2022). With falls as the leading cause of preventable death and hospitalizations, it became vital for in-person evidence-based falls prevention programs (EBFPPs) to pivot to remote delivery. EBFPPs are community-based programs that have demonstrated effectiveness through rigorous research to prevent falls and have standardized protocols and trained facilitators to disseminate and deliver the program (U.S. Department of Health and Human Services & Administration for Community Living, 2021). While some evidence-based programs were offered successfully via remote delivery prior to the pandemic, transition to remote delivery across the evidence-based program community was limited due to funding constraints and the time needed for rigorous effectiveness testing of the remote modality (Jaglal et al., 2013; Lorig et al., 2006, 2008). These barriers to remote delivery included perceived difficulty related to technology required, perceived lack of interest among instructors and participants, lack of funding to support transition to remote delivery, and lack of support from funders for remote delivery without extensive research on effectiveness (Gray et al., 2022; Kahlon et al., 2021; Li et al., 2021; Patel et al., 2021).

In Spring 2020, many EBFPP administrators began re-designing programs for remote delivery to accommodate physical distancing guidelines necessitated by the pandemic (Vincenzo et al., 2021). Transition to remote delivery was essential for older adults and persons with disabilities to access EBFPPs for staying healthy, falls and injury free, and out of hospitals, but also keeping them socially engaged. We collaborated with the Administration on Community Living (ACL), the National Council on Aging (NCOA), and the National Falls Prevention Resource Center (NFPRC), for in-depth implementation evaluation of remotely delivered EBFPPs. While the need to adapt EBFPPs that were designed and tested in in-person settings in response to COVID-19 was clear, the best process for making those adaptations and implementing them with fidelity was not.

We examined the process of adapting and implementing four EBFPPs for remote delivery at the program and delivery-site level, and best practices for implementing the programs remotely within the RE-AIM framework (Glasgow et al., 2019). The objective was to identify elements necessary for programs to adapt to remote delivery with fidelity to maintain acceptability, feasibility, and sustainability within community-based delivery settings. This enhances NFPRC’s ongoing work supporting dissemination, implementation and sustainability of EBFPPs. Participant-level effectiveness and implementation outcomes were beyond the scope of this funding, and are being evaluated under separate funding (Steinman et al., 2022).

## Methods

This was a qualitative evaluation study at the program and delivery-site level. Qualitative data focused on answering questions about the adaptation and implementation process for remote delivery.

### Participants and Setting

Four EBFPPs participated in the evaluation: A Matter of Balance (AMOB, (MaineHealth, 2022)) is a group-based education and exercise program delivered in nine, 2-hour sessions designed to reduce fear of falling and increase activity levels; EnhanceFitness (EF, (Project Enhance, 2022)) is a group-based exercise program which includes three, 1-hour sessions for at least 16 weeks, and focuses on improving upper and lower extremity strength, balance, and cardiovascular fitness; Stepping On (SO, (Wisconsin Institute for Healthy Aging, 2022)) is a group-based education program designed to address multiple factors influencing falls, and is delivered in seven 2-hour sessions; and Tai Ji Quan – Moving for Better Balance (TJQ-MBB, (Tai Ji Quan: Moving for Better Balance, 2016)) is a group-based balance training program based in contemporary Tai Ji Quan, and is delivered in two 1-hour sessions per week for at least 24 weeks.

Participants were drawn from two groups. The first group were program administrator representatives. Program administrators are responsible for licensing, training, and supporting delivery sites. They are also the deciding authority on whether or what changes or adaptions may be made to the content or curriculum of the program. Adaptations made or delivered without the approval of the program administrator are considered out of fidelity with the evidence-base. One administrator representative from each of the four programs participated.

The second group were program staff and leaders. Program staff oversee the operational and administrative elements of program delivery at delivery sites, such as scheduling, recruiting, and data collection. Program leaders are trained instructors eligible to deliver the program at delivery sites. In January 2021, an online survey was sent out through the four program administrators and existing program staff and leader networks to gauge evaluation participation interest. Respondents provided information about programs offered remotely, populations served by their programs, and interest in participation. We purposively sampled for sites offering one of the four EBFPPs remotely and that served rural, BIPOC and/or disability communities. The UW team recruited interested sites by phone and email to confirm remote delivery, class timelines, expected enrollment, and ability to collect participant data. The enrollment target was 50 participants per program, and 200 participants total.

### Eligibility Criteria

Participating delivery sites had to meet the following eligibility criteria: offering one of the four EBFPPs remotely; remote class offering starting after February 15, 2021 and ending by September 30, 2021, enrolling new participants. Programs ending after September 30, 2021 were not eligible to participate due to the funding timeline.

### Data collection

#### Qualitative Data

The four program administrator representatives participated in two rounds of interviews in January 2021 and January 2022. Informed by the FRAME adaptations framework (Wiltsey-Stirman et al., 2019) and Rabin et al.’s assessment of adaptations (2018), our interview guide solicited information about what, how, and why adaptations were made. Additional questions informed by the RE-AIM and Implementation Outcomes Frameworks (IOF) focused on equity and asked about reach-the number and representativeness of individuals willing to participate (Proctor et al., 2011; Shelton et al., 2020); accessibility-the perceived fit or compatibility of the innovation for delivery organizations and instructors; feasibility-the actual fit or suitability of the innovation for delivery organizations and instructors; costs-the monetary and non-monetary costs of implementing the innovation; and impacts of adapting EBFPPs to remote delivery for program administrators and delivery sites. Utilizing these frameworks collectively provided for more comprehensive evaluation of questions of interest. Rabin’s simplified approach to FRAME distilled adaptations into the intuitive domains of what, how, and why to better classify and organize adaptations; this was contextually important to this evaluation to capture how EBFPPs were adapted to remote delivery in chaotic circumstances. IOF looks at key outcomes needed for remote delivery to be successful, and those outcomes are hypothesized to positively impact participant health and behavioral outcomes; measuring feasibility and accessibility is important in its own right and also in understanding the pathway to preventing falls through new delivery modes. RE-AIM, as an evaluation framework, moves beyond effectiveness to explore the public health impacts of programs, and compliments Rabin’s domains by understanding why adaptations were made and the impact of those adaptations.

EBFPP staff and leaders (*n* = 19) were interviewed in August and September 2021 using the same interview guide as program administrators/developers. While the questions were the same, the perspective from the staff and leaders focused on how the adaptations made by the administrators were implemented at the program delivery-site level. We invited staff and leaders who successfully delivered EBFPPs remotely, as well as staff and leaders who had intended to deliver remote EBFPPs but did not, to guide both best practices and understand what barriers to delivery need to be addressed going forward.

#### Data Analysis

Interviews were analyzed using deductive thematic analysis and a rapid analysis approach (Gale et al., 2019; Nowell et al., 2017; Sandelowski et al., 2009). Deductive thematic analysis is a method for identifying, analyzing, organizing, and reporting themes in qualitative data that can be trusted and confirmed by others using the same method; rapid analysis is a method of summarizing verbatim transcripts in to a structured matrix that consolidates information into pre-determined theme categories. The codebook was generated using FRAME, IOF, and RE-AIM. A subset of interviews was double-coded by two team members and compared for agreement. Coding discrepancies were discussed to achieve coding consensus. Remaining interviews were coded by one team member.

## Results

Across the four EBFPPs, 16 sites enrolled in the evaluation. Eleven of 16 sites successfully held their remote EBFPP sessions as scheduled. Among the five sites that enrolled but did not deliver their classes as scheduled, reasons for classes not being delivered varied: classes rescheduled to a later date outside of the evaluation period; cancelled due to low enrollment; or no longer offering EBFPP remotely. Delivery sites and participant enrollment are summarized in Table 1. One hundred and fifty-two (152) new participants were enrolled across the four EBFPPs. Enrollment by program ranged from 14 to 68 participants; only TJQ-MBB met the target of 50 enrolled participants.

**Table 1:** Delivery sites and participants enrolled

Program	Delivery sites enrolled	Delivery sites completing classes	Participants enrolled
A Matter of Balance	3	2	14
EnhanceFitness	5	3	41
Stepping On	5	3	29
Tai Ji Quan – Moving for Better Balance	3	3	68

## Approach to Adaptations

In January 2021, we interviewed the main representative from each of the four EBFPP programs to better understand adaptations made to their programs. While the actual adaptations varied by program, and adaptations needed for physical activity-based programs were somewhat different than those needed for education-based programs, there were many thematic similarities in the approach to adaptations.

### FRAME Adaptations Framework and Assessment of Adaptations

#### How Were Adaptations Made?

Adaptations were an iterative and collaborative process. While the final decisions about the adaptations were made by the program administrators/developers, they included a broad array of stakeholders in the process of determining and defining needed adaptations for remote delivery. Stakeholders participating in the process included program participants, leaders/instructors, trainers, delivery organizations, researchers, other evidence-based program administrators, professional clinical organizations, advocacy organizations, and funders. For all programs, after initial adaptations were identified, they were pilot tested and evaluated with a subset of delivery organizations; revisions were then made based on pilot feedback.

We partnered with the folks at [the health system] to help fund and support a couple of pilots around remote adaptations using Zoom or some other kind of remote video and audio platform…and part of our staff worked hand-in-hand with them to go through both the training components of that program as well as the curriculum to sort of put together a proof of concept as to whether people could be trained to deliver Matter of Balance remotely via Zoom, and--if they could--what kind of adaptations had to be done to the program in terms of safety and fidelity and engagement. - Program staff [101]

#### Why Were Adaptations Made?

First and foremost, the need for EBFPPs continued despite the advent of COVID-19; indeed, the need for EBFPPs may have increased as a result of reduced access to in-person programming and increased sedentary behavior during COVID lockdowns. It was absolutely necessary to ensure EBFPPs were accessible remotely when they could not be offered in person.

Second, participant safety was the primary driver guiding adaptation decisions. All four participating EBFPPs cited safety as a concern, and safety was a recurrent theme whether the adaptation related to program content, program context, or program training and evaluation.

Third, adaptations were selected based on priority. Incremental adaptation allowed for prioritizing critical elements while also ensuring the adaptations were acceptable and feasible for delivery organizations. Iterative adaptation allowed developers to continue to work collaboratively with stakeholders to incorporate feedback and hone adaptations in ways that both maintained fidelity to the evidence-based model while also supporting feasibility for delivery organizations to implement the adaptations.

So for [our state]…We were paused quite early in all of this, we were paused right away in March [2020]. We had in-process research projects going on…so we worked with researchers…to look at remote delivery so that the study could continue…So we had a little actual delivery experience that gave us the initial traction for the direction we wanted to go in…We sat down with the manual and the master trainers and thought through…wanting to make sure it was safe, wanting to make sure it was interactive. – Program administrator [2]

#### What Adaptations Were Made?

**Content** For the physical activity programs (EF and TJQ-MBB), some specific adaptations were needed to address physical-activity content components in a remote setting where instructors were not able to offer feedback on form or function in the same way. As noted above, these content changes were guided by participant safety. Adaptations included providing options for exercises to be done while seated rather than standing, and contextualizing the movements in space that can be difficult to see on screen such as instructors offering demonstrations from both a front and side view for participants. For TJQ-MBB, some content was eliminated for safety reasons for remote programming, such as activities usually done with eyes closed. Some adaptations to content resulted specifically from the move to remote delivery. For example, TJQ-MBB includes an activity where participants are all walking in a confined space to practice navigating crowded settings; SO includes a session where participants can try out a variety of mobility aids, such as walking sticks. Neither of these is feasible in a remote setting and were removed from the remote curriculum.

They changed some of the exercises, so they removed some that could only really be done standing, so the exercises that were performed in the [remote] workshop were just done seated, which I felt was a really wise move, considering it's a fall prevention class. – Program manager and leader [105]

Other content across physical activity and behavioral education programs remained largely the same, in line with the original models of how the programs were developed, tested, and proven effective. Minor content adaptations included instructors and leaders pausing more frequently or taking more breaks.

##### Context

Safety was also a theme of context adaptations, or modifications to the way the program was delivered. Leaders/instructors had participant contact information, emergency contact information and participant physical location in case of any emergency during the remote session. Other context adaptations supported the use of remote technology. This included a “Session Zero,” or a kind of test-run before the class so that participants could trouble-shoot any technology issues or ask questions, and including time for participants to check space and equipment for safety prior to the start of class. Some programs also included additional personnel as a monitor for remote classes. Some monitors were available just at the beginning of class to support technology needs. For remote-delivered EF, a second trained instructor is required throughout the class to monitor safety. Minor adaptations to context included opening the remote platform before the start of class, or leaving it open after the end of class to allow for social connection among participants.

We have kind of a pre-practice class with each individual person. So I set up just like a practice meeting with them to run through all of those technological things and then just make sure that the space that they're going to be doing the class in is safe, and that I can see them, you know from the head down to the floor when they're doing the exercises… - Program leader [103]

Based off of [the program developer’s] guide that they came out with this or that, you needed to have two [instructors]. Basically, one to do the exercises and one to be watching, and that makes a whole lot of sense. It's hard to see, you know, on a little computer screen what's going on. - Program staff [117]

##### Training

All programs transitioned their instructor/leader trainings to remote format during the pandemic. Requirements for additional training for remote delivery varied by program, with some EBFPPs encouraging existing instructors/leaders to participate in remote delivery training and others requiring supplemental training before instructors/leaders could deliver the program remotely. Training related to adaptations for remote delivery fell into two categories: training related to the content changes for delivering the curriculum safely and with fidelity in a remote format, and training specific to utilizing the remote platform. The latter included training about using various remote platforms to ensure leaders/instructors were facile using the technology on their own and to support class participants, and engagement and facilitation skills for remote delivery that differ from in-person delivery.

The virtual training that we had was…- how do you present virtual classes… It was what do you need to do to, … how do you demonstrate the exercises virtually so that the participants can see you, and that you can still be safe and see the participants …. – Program leader [103]

*…*we have lost a couple of instructors, so having that remote training option for instructors and not necessarily needing to have a master trainer available to come and do the training or sending people away [to training], that's really helped. – Program staff [119]

##### Documentation

All programs developed written guidance summarizing the adaptations for remote delivery and requirements for remote delivery training. Written guidance was developed collaboratively, similar to the process for developing the adaptations, and program administrators and developers solicited input and incorporated feedback from stakeholders into final guidance documents.

#### Reviewing Adaptations After Initial Remote Delivery Implementation

In January 2022, we interviewed the four EBFPP program administrators/developers a second time to gather information about how, if at all, the initial adaptations, training, and guidance created and implemented in 2020 and early 2021 changed over the course of implementation throughout 2021. EBFPP administrators/developers reported that there were not any material changes to the adaptations, training or guidance documents from what was originally developed. They did provide additional clarifications as needed to address specific questions, or better describe the adaptations.

## RE-AIM Framework

### Reach

#### Who Is Reached with Remote Delivery That Was not Reached With In-person Programming?

EBFPP administrators and leaders reported that remote delivery reached people who previously experienced barriers to in-person programming due to limitations in transportation, those who lived far from an in-person site including rural participants, and those who were more homebound due to physical limitations. Leaders also noted remote delivery was reaching caregivers, and made the program more available in places with cold winter climates where weather can make travel to in-person programming challenging or unsafe.

It's great because it does remove the barriers of transportation and people who have caregiving responsibilities. – Program staff [116]

Low population areas where there’s not usually enough demand to do programs as routinely as they might need to be done to keep people engaged, we’re seeing a lot of that. – Program staff [101]

#### Who Is not Being Reached with Remote Delivery?

Both EBFPP administrators and leaders noted the digital divide as the primary barrier to reaching participants through remote delivery. Barriers linked to the digital divide included lack of access to appropriate devices for participating in remote EBFPPs; lack of internet infrastructure to support video streaming platforms like Zoom, especially in rural areas without broadband internet; and lack of skills among participants, and sometimes leaders, to use the devices and digital delivery platforms. Leaders also noted that remote programming didn’t reach some groups of new participants, particularly traditionally underserved communities, including immigrant communities, communities of color, communities speaking a primary language other than English, and some disability communities such as people with vision limitations for whom a tablet or laptop screen is insufficient to participate. Leaders also noted attitudinal barriers among older adult participants, including participants preferring in-person over remote, discomfort with computer technology in general, or not wanting other participants to see inside their homes on video.

I really think that a lot of people in lower socio economic situations do not have access to the internet or computers…and when we started the remote program I told them that we were going to be doing this and they don't have access…they don't have the means to participate…I could give them the weights and get chairs if they needed, but as far as a computer, or even knowing how to use a computer, or having a access to the internet, that is limiting I think for a lot of people. – Program staff and leader [118]

## Implementation Outcomes Framework

Interviews with EBFPP administrators/developers, delivery site staff, and instructors/leaders also assessed a number of implementation outcomes, including accessibility, feasibility, and cost all centered within the context of equity.

### Accessibility

We considered accessibility at two levels—delivery organization and leader. EBFPP administrators reported that programs were accessible to delivery organizations, particularly those who had previously delivered the programs in-person. The barriers to accessibility at the organization level reflected the context of the pandemic. Many community-based organizations had to shift priorities toward COVID response and basic needs, and away from EBFPPs and other preventive programing; only a fraction of pre-pandemic classes shifted to remote delivery. In addition, periods of lockdown early in the pandemic resulted in many staff being furloughed or laid off; this limitation in the workforce further impacted the ability to bring EBFPPs back through remote delivery as organizations may not have had the staff or leaders to organize or deliver the programs.

Among leaders, accessibility to continue leading programs was mixed. Not all leaders had necessary devices or sufficient internet access to teach from their homes. Some delivery organizations were able to provide devices or support internet costs for their leaders, but not all. Some leaders were not comfortable using devices or remote delivery platforms in general, let alone to lead a class. Administrators tried to support leaders to learn and use the technology through training and ongoing technical support. Offering both technical support for leaders to utilize remote delivery technology and support for best practices in online learning and engagement will improve accessibility at the delivery site and leader levels.

But I can say I've had some instructors who have been teaching for 20 years but were not comfortable with virtual. The whole technology part of it, you know, the way it just they felt was a bit more impersonal. – Program staff [114]

### Feasibility

Feasibility reflects what supports or impedes the delivery of remote programming. EBFPP administrators reported that remote delivery was generally feasible. However, they also noted that remote delivery is more resource intensive compared to in-person delivery, with extra time required to send materials to participants that otherwise would have been distributed in person, extra staff required to ensure safety for some programs, and new needs to support technology. While grant funds are available to support remote-delivered EBFPPs, those grants often do not cover the additional resources required to launch or maintain remote programming, such as a leader who needs a wireless microphone. None of the programs required delivery sites to use a single platform, such as Zoom. While this provided flexibility to delivery sites, it also introduced challenges to select a platform, train users and leaders to use the platform, or manage support for the platform across different hardware interfaces (e.g., Android vs. iPhone).

…we would call and also email, schedule the session zero, not everyone would show up on the scheduled day and time, so having to hunt people down to get them set up before class…Getting the book sent out to them, getting their paperwork back signed, if one page was illegible you know there's a lot of follow up, whereas in person we're like, “Hey you didn't check the box, here's the form, check the box give it back to me.”…it's a lot more difficult to get the required paperwork from people online. – Program manager and leader [105]

### Cost

The increased resource intensity noted above translated into increased costs for remote delivery. Additional staff to monitor classes for safety, staff time to send materials to participants and provide technology support, mailing costs, and technology costs for devices and accessories, improved internet speed or capacity, and remote platform licenses all increased the costs to deliver the program. While sites did not incur costs such as renting space or providing snacks, these savings did not offset the cost increases noted above.

[The cost has] gone up. If there was just the one instructor, it would obviously be less, but since there's another…that adds to the cost. – Program staff and leader [118]

The costs have gone up dramatically…Both in terms of training and keeping staff engaged when they’re pulled in many other different directions. In terms of the recruitment strategies that we have to have in order to get people to sign up for a class, where normally…we could just grab and tap them on the arm in the senior center and say, “Oh my gosh, this program starts next week, you have to go.”…In terms of the program delivery, like I said earlier, now we have three people instead of normally two staff for managing the programs. The data collection takes considerably longer, because we’re chasing data much more than before when we could just set people down for ten minutes at a workshop and ask them to complete the program. – Program staff [101]

### Impact

Impacts of the transition to remote delivery include positive impacts, but also challenges, barriers and unintended consequences. Technology capacity and efficacy improved at the organization level with delivery organizations learning and leveraging new hardware and software for remote delivery, data collection and technical support; capacity and efficacy also improved at the individual level for leaders who learned to use new hardware and software, and could use those skills for other remote interactions besides program delivery. In a time when many public settings were closed, remote delivery offered a forum for social connection. Going forward, delivery organizations and leaders noted remote delivery as an opportunity to improve reach, accessibility and sustainability.

Some programs experienced improved adherence when participants did not have to travel to attend in-person, but other delivery organizations and program leaders experienced increased no-shows or participants dropping out early in the program. Additional support and access to hardware was not always enough for some leaders to feel comfortable using the technology to deliver remote programming. For these leaders, many are waiting for in-person programming to return to re-engage in instructing but this has been slow. While many delivery organizations were able to find resources to provide access to devices and internet connectivity for leaders that needed them, resources were very limited, they could not provide devices to everyone, or overcome broadband infrastructure limitations, all of which may limit accessibility among delivery sites and leaders.

## Discussion

We conducted a qualitative study evaluating remote-delivery implementation among four EBFPPs, drawing on the experiences of program administrators, delivery organizations, program leaders and program staff. Not all sites recruited were able to successfully deliver their remote programming. Among delivery sites that did deliver their remote programs as part of this evaluation, 152 new participants were enrolled. Among sites that were unable to successfully delivery their planned remote programming, reasons reflected the shifting social context of the pandemic during mid-2021. During this time, vaccines were becoming widely available, with most older adults receiving their vaccines in early 2021. As a result, planned remote classes for May through September 2021 received less interest as more people were vaccinated and delivery sites and participants sought a return to in-person programming.

EBFPPs made a variety of adaptations in the transition to remote delivery. Adaptations were made collaboratively with stakeholders, pilot tested, and revised iteratively as needed. Adaptations were intended to address the ongoing need for EBFPPs during COVID-19 lockdowns, ensure safety for participants, and be feasible for delivery organizations and program leaders. Adaptations included changes to program content, context, and training. All adaptations were documented by program administrators and shared with delivery organizations, trainers, and program leaders.

Remote delivery impacted program reach and implementation. While some new communities were reached that previously experienced barriers to in-person programming, the digital divide remained a primary barrier to reaching communities most in need and accessibility for leaders and delivery sites struggling with remote delivery. Accessibility, feasibility and cost are important factors to sustainability of remote-delivered EBFPPs. Delivery organizations had to shift their priorities in response to the pandemic, and not all were able to continue offering EBFPP in any format. For those that could transition to remote delivery, staff capacity, leader availability, access to appropriate hardware, software, and Internet, and costs impacted feasibility. Remote delivery is more resource intensive and incurs increased costs despite some savings compared to in-person delivery. Among leaders, accessibility was mixed and primary barriers were technology related.

**Fig. 1:**
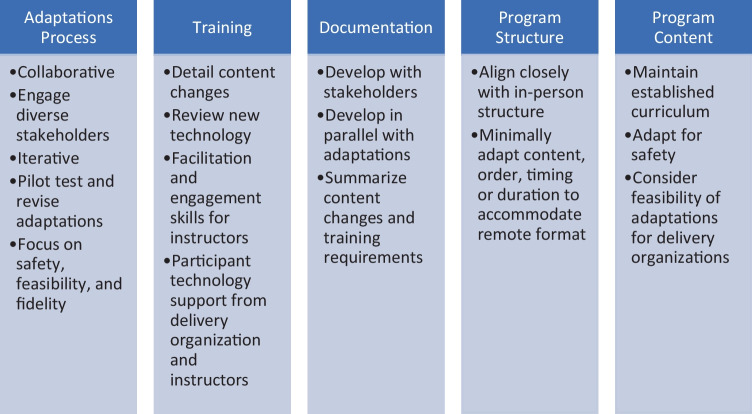
Recommended approaches for adapting to remote delivery

**Fig. 2:**
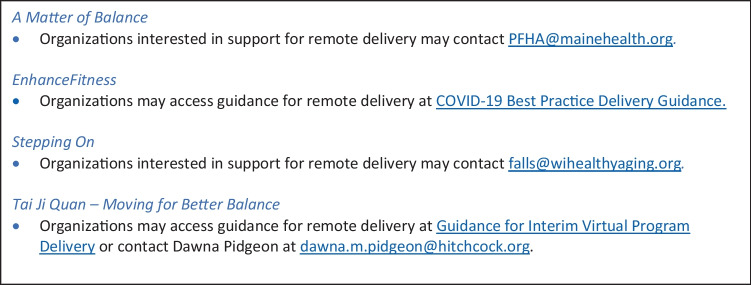
Resources for remote EBFPP implementation

Remote delivery will continue beyond the pandemic despite some of the challenges and barriers because returning to only in-person programming may eliminate access for some underserved communities, including caregivers, people with disabilities, immunocompromised people, and those who prefer remote delivery. As one program administrator noted, “The remote delivery genie is out of the bottle, and it can’t go back.” However, it is also critical that adaptations for remote delivery are made consistent with the existing evidence base to ensure continued fidelity and effectiveness. Findings from this study can inform funders and program administrators about the remote delivery adaptations process to ensure consistency with existing evidence, and maintaining fidelity in implementation. Recommended approaches to developing, documenting and implementing adaptations with fidelity in alignment with the evidence are summarized in Fig. 1. Organizations that would like support for implementing remote programming can access support and resources through program administrators and program websites (Fig. 2).

Adaptations were developed, pilot tested, revised, and implemented expediently in response to COVID-19 lock-downs to ensure fall-prevention programming remained available. Despite being in our third year living with COVID-19, the traditional approach to advancing the evidence-base through randomized control trials would not have proceeded quickly enough to be responsive to the need for remote delivery (Miller et al., 2021; Wiltsey-Stirman et al., 2019; von Thiele Schwarz et al., 2019). To be sure, even if randomized trials could have been funded and launched in 2020 to assess implementation of remote delivery, we would only now likely be completing those trials and starting to understand how it compared to the in-person evidence base, leaving people in need of fall prevention programming without a viable option while in-person programming was restricted. This study helps demonstrate that established programming can be responsive to emergency situations, leverage the stakeholder networks, and adapt to shifting realities in ways that sustain programming availability, maintain fidelity, and adhere to established evidence and protocols within the situational context.

Findings from this evaluation have implications for both maintaining programs and for policies needed to support remote program delivery and sustainability. Remote program delivery aligns with several of the eight factors for sustainability identified in the Program Sustainability Framework: adaptations, organizational capacity, and funding (Schell et al., 2013). This evaluation demonstrated that in-person EBFPPs could be adapted to remote delivery, ensuring that key program content could be delivered via video-conferencing so that organizations, leaders, and older adults maintained access to health promotion programming. EBFPP program administrators and community-based delivery organizations built capacity for remote training and delivery, providing opportunities to not just maintain but expand the workforce and program offerings. Adapting to remote delivery may also improve access to populations traditionally underserved by these falls prevention programs, such as people living in rural areas and people living with disabilities. Modifying Research Tech Age’s *Guidelines for Delivering Telewellness Programs to Older Adults with Disabilities* for evidence-based falls prevention programs would offer specific guidance on planning for and delivering remote classes (Mitzner et al., 2021).

Older adult advocates are asking for increases in funding for ACL Falls Prevention state and local grant funding and the promised—but not yet funded—Research, Demonstration, and Evaluation Center for the Aging Network (National Council on Aging & Aging and Disability Business Institute, 2021). Both policies provide essential and needed support for our growing aging population, and continuing both research and practice to improve access, quality, and cost of falls prevention programming for older adult health equity. Evaluation findings suggest that while remote program delivery can improve reach and access, additional costs are incurred by community-based delivery organizations that engage underserved communities but are not equipped for remote delivery. While some grant funding mechanisms were adjusted during the pandemic to cover costs specific to remote delivery such as hardware and software, it was not always sufficient to cover the new or additional costs associated with remote delivery. Furthermore, the policymakers are being asked to continue funding for tele-health and other remote-delivered services that were previously not covered pre-COVID, whether through grant mechanisms, reimbursement, or coverage from federal, state, or local government and private payors. While remote delivery allows for falls prevention class providers to be located in different geographic areas than class participants, it is likely they will need to remain in the same region or state until there is parity across telehealth laws throughout the U.S. Collaborating across public health, aging, social service, and health care sectors on continued remote EBFPP coverage will provide continuity of care in older adults’ homes and communities.

This study had a number of strengths and limitations. Qualitative data was gathered from program administrators, leaders, and staff, offering broad and deep insight on the adaptation and implementation of remote-delivered EBFPPs. Challenges to data collection for both quality improvement and program evaluation were exacerbated by the shift from in-person to remote delivery where collecting data became a more burdensome task for delivery organizations. This includes the ability to determine how many organizations were possibly eligible to participate. Between 2014 and March 2020, over 10,000 falls prevention program workshops were delivered across hundreds of sites; limitations in administrative records preclude the ability to know exactly how many sites delivered EBFPPs remotely. While we estimate the number of eligible sites numbered in the hundreds, we cannot verify this; we also recognize that the 16 organizations that agreed to participate is a small fraction of potentially eligible sites, and may differ from sites that did not participate. The proposed Research, Demonstration, and Evaluation Center for the Aging Network provides an opportunity to streamline and support data collection to understand the impact and value of remote delivered EBFPPs (Menne, 2022). In addition, we tried to recruit and enroll sites to achieve maximum variability on communities and populations served, including BIPOC, disability, limited English proficiency, low-income, and rural communities; there may have been self-selection bias among sites that agreed to participate in the evaluation, and participating sites may differ from non-participating sites. However, we did receive both support for and critiques of the programs during interviews, reflecting a balance in perspectives, or possibly an increased likelihood of interview engagement among staff or leaders with negative experiences.

Future research can advance this work in a number of ways. First, other evidence-based programs (EBPs) may use the approach described here to develop, implement, and evaluate adaptations for remote delivery (Gell et al., 2021). Second, effectiveness studies should measure the participant outcomes for remote delivery and compare to in-person delivery for EBFPPs and other EBPs. Some of this work is currently underway (Patel et al., 2021). Third, implementation studies should explore participant acceptability of remote versus in-person delivery, and whether remote delivery impacts acceptability as measured by attendance and adherence which could—in turn—impact participant outcomes. Last, adaptations should be tracked and documented on an ongoing basis to assess the trajectory of adaptations over time, and impacts within the RE-AIM and FRAME-IS frameworks to address equity and sustainability.

## Conclusion

This study evaluated adaptation development, documentation and implementation for four EBFPPs. Findings demonstrate remote EBFPPs made planned and fidelity-consistent adaptations to remote delivery in partnership with researchers and community organizations, focusing on participant safety both in program content and delivery. Supports using and accessing technology were needed for participants and leaders to facilitate engagement, and improved over time. While remote EBFPP delivery has increased access to EBFPPs for some populations (e.g. caregivers, rural-dwellers, persons with physical disabilities), the digital divide remains for some underserved communities in access to and comfort using technology. Remote-delivered EBFPPs were acceptable and feasible for delivery organizations and leaders and were able to be delivered with fidelity using adaptations from program developers, but were more resource intensive and costly to implement compared to in-person.

This work has important implications beyond the pandemic. Remote delivery has expanded access to groups traditionally underserved by in-person programming, particularly disability communities. Funders can ensure this expanded access to programming via remote delivery remains by supporting a structured approach to developing, documenting, and implementing adaptations to established EBFPPs and other EBPS; treating remote-delivered EBFPPs and EBPs that follow this approach to adaptations the same as those delivered in-person; and funding continued study and evaluation of remote-delivered programs to expand and reinforce the existing evidence.

## Data Availability

De-identified interview transcripts may be requested from the corresponding author.

## References

[CR1] Gale RC, Wu J, Erhardt T, Bounthavong M, Reardon CM, Damschroder LJ, Midboe AM (2019). Comparison of rapid vs in-depth qualitative analytic methods from a process evaluation of academic detailing in the Veterans Health Administration. Implementation Science.

[CR2] Gell, N., Hoffman, E., & Patel, K. (2021). Technology Support Challenges and Recommendations for Adapting an Evidence-Based Exercise Program for Remote Delivery to Older Adults: Exploratory Mixed Methods Study. *JMIR Aging*, *4*(4), e27645. 10.2196/27645 10.2196/27645 PMC8704113 34889743

[CR3] Glasgow, R. E., Harden, S. M., Gaglio, B., Rabin, B., Smith, M. L., Porter, G. C., Ory, M. G., & Estabrooks, P. A. (2019). RE-AIM Planning and Evaluation Framework: Adapting to New Science and Practice With a 20-Year Review. *Frontiers in Public Health*, *7*. https://www.frontiersin.org/article/10.3389/fpubh.2019.00064 10.3389/fpubh.2019.00064 PMC6450067 30984733

[CR4] Gray SM, Franke T, Sims-Gould J, McKay HA (2022). Rapidly adapting an effective health promoting intervention for older adults—choose to move—for virtual delivery during the COVID-19 pandemic. BMC Public Health.

[CR5] Hoffman, G. J., Malani, P. N., Solway, E., Kirch, M., Singer, D. C., & Kullgren, J. T. (2022). Changes in activity levels, physical functioning, and fall risk during the COVID-19 pandemic. *Journal of the American Geriatrics Society*, *70*(1), 49–59. 10.1111/jgs.17477 10.1111/jgs.17477 34536288

[CR6] Jaglal SB, Haroun VA, Salbach NM, Hawker G, Voth J, Lou W, Kontos P, Cameron JE, Cockerill R, Bereket T (2013). Increasing Access to Chronic Disease Self-Management Programs in Rural and Remote Communities Using Telehealth. Telemedicine Journal and E-Health.

[CR7] Kahlon MK, Aksan N, Aubrey R, Clark N, Cowley-Morillo M, Jacobs EA, Mundhenk R, Sebastian KR, Tomlinson S (2021). Effect of Layperson-Delivered, Empathy-Focused Program of Telephone Calls on Loneliness, Depression, and Anxiety Among Adults During the COVID-19 Pandemic: A Randomized Clinical Trial. JAMA Psychiatry.

[CR8] Li F, Harmer P, Voit J, Chou L-S (2021). Implementing an Online Virtual Falls Prevention Intervention During a Public Health Pandemic for Older Adults with Mild Cognitive Impairment: A Feasibility Trial. Clinical Interventions in Aging.

[CR9] Lorig KR, Ritter PL, Dost A, Plant K, Laurent DD, Mcneil I (2008). The expert patients programme online, a 1-year study of an Internet-based self-management programme for people with long-term conditions. Chronic Illness.

[CR10] Lorig, K. R., Ritter, P. L., Laurent, D. D., & Plant, K. (2006). Internet-Based Chronic Disease Self-Management: A Randomized Trial. *Medical Care*, *44*(11). https://journals.lww.com/lww-medicalcare/Fulltext/2006/11000/Internet_Based_Chronic_Disease_Self_Management__A.2.aspx 10.1097/01.mlr.0000233678.80203.c1 17063127

[CR11] MaineHealth. (2022). *A Matter of Balance*. https://www.Mainehealth.Org/Healthy-Communities/Healthy-Aging/Matter-of-Balance

[CR12] Menne, H. (2022). Difficulties demonstrating impact of OAA programming and glimmers of hope. *Generations*, *45*(4).

[CR13] Miller CJ, Barnett ML, Baumann AA, Gutner CA, Wiltsey-Stirman S (2021). The FRAME-IS: A framework for documenting modifications to implementation strategies in healthcare. Implementation Science.

[CR14] Mitzner, T. L. T., Mitzner, T. L., Remillard, E., Cohen, K., & Cochran, L. (2021). *TechSAge Tool: Guidelines for delivering telewellness programs to older adults with disabilities*. Rehabilitation Engineering Research Center on Technologies to Support Aging-in-Place for People with Long-Term Disabilities. https://techsage.gatech.edu/sites/default/files/2021-08/TechSAge%20Tool_Telewellness%20Guidelines_V1_Final.pdf

[CR15] National Council on Aging, & Aging and Disability Business Institute. (2021). *Policy Spotlight: Aging Network Opportunities in the New Older Americans Act Research, Demonstration and Evaluation Center*. https://2yjszzobx7o304u1b45x6bsd-wpengine.netdna-ssl.com/wp-content/uploads/2021/05/Policy-Spotlight-OAA-Research-FINAL-508.pdf

[CR16] Nowell LS, Norris JM, White DE, Moules NJ (2017). Thematic Analysis: Striving to Meet the Trustworthiness Criteria. International Journal of Qualitative Methods.

[CR17] Patel, K. V., Hoffman, E. V., Phelan, E. A., & Gell, N. M. (2021). *Remotely Delivered Exercise to Rural Older Adults with Knee Osteoarthritis: A Pilot Study*. 10.20944/PREPRINTS202107.0433.V1 10.1002/acr2.11452 PMC9374047 35687577

[CR18] Proctor E, Silmere H, Raghavan R, Hovmand P, Aarons G, Bunger A, Griffey R, Hensley M (2011). Outcomes for implementation research: Conceptual distinctions, measurement challenges, and research agenda. Administration and Policy in Mental Health and Mental Health Services Research.

[CR19] Project Enhance. (2022). *Enhance Fitness*. https://projectenhance.org/enhancefitness/

[CR20] Quan, T. J. Moving for Better Balance. (2016). *About the program. *Retrieved from: https://tjqmbb.org/

[CR21] Rabin BA, Burke RE, Hess PL, McCreight M, Ayele R, Battaglia C, Glasgow RE, Frank JW (2018). Systematic, Multimethod Assessment of Adaptations Across Four Diverse Health Systems Interventions. Frontiers in Public Health.

[CR22] Sandelowski M, Voils CI, Knafl G (2009). On Quantitizing. Journal of Mixed Methods Research.

[CR23] Schell SF, Luke DA, Schooley MW, Elliott MB, Herbers SH, Mueller NB, Bunger AC (2013). Public health program capacity for sustainability: A new framework. Implementation Science.

[CR24] Shelton RC, Chambers DA, Glasgow RE (2020). An Extension of RE-AIM to Enhance Sustainability: Addressing Dynamic Context and Promoting Health Equity Over Time. Frontiers in Public Health.

[CR25] Steinman, L., Fall Creek, S., & Lorig, K. (2022, March 2). Evaluation for Equity: Delivering Remote Health Promotion During COVID. *Workshop Presentation at the 2022 Society for Public Health Educators (SOPHE) Annual Meeting (Virtual)*.

[CR26] Wiltsey-Stirman S, Baumann AA, Miller CJ (2019). The FRAME: An expanded framework for reporting adaptations and modifications to evidence-based interventions. Implementation Science.

[CR27] U.S. Department of Health and Human Services, & Administration for Community Living. (2021, July 21). *Health Promotion: ACL Definition of Evidence-Based Programs*. https://acl.gov/programs/health-wellness/disease-prevention#future

[CR28] Vincenzo, J. L., Hergott, C., Schrodt, L., Rohrer, B., Brach, J., Tripken, J., Shirley, K. D., Sidelinker, J. C., & Shubert, T. E. (2021). Capitalizing on Virtual Delivery of Community Programs to Support Health and Well-Being of Older Adults. *Physical Therapy*, *101*(4), pzab001. 10.1093/ptj/pzab001 10.1093/ptj/pzab001 PMC8023634 33439254

[CR29] von Thiele Schwarz U, Aarons GA, Hasson H (2019). The Value Equation: Three complementary propositions for reconciling fidelity and adaptation in evidence-based practice implementation. BMC Health Services Research.

[CR30] Wisconsin Institute for Healthy Aging. (2022). *Stepping On*. https://wihealthyaging.org/programs/falls-prevention-programs/stepping-on/

